# Feasibility of the virtual reality-based assessments in patients with panic disorder

**DOI:** 10.3389/fpsyt.2023.1084255

**Published:** 2023-01-24

**Authors:** Byung-Hoon Kim, Jae-Jin Kim, Jooyoung Oh, Seung-Hyun Kim, Changsu Han, Hyun-Ghang Jeong, Moon-Soo Lee, Junhyung Kim

**Affiliations:** ^1^Department of Psychiatry, Yonsei University College of Medicine, Seoul, Republic of Korea; ^2^Institute of Behavioral Sciences in Medicine, Yonsei University College of Medicine, Seoul, Republic of Korea; ^3^Department of Psychiatry, Korea University Guro Hospital, Korea University College of Medicine, Seoul, Republic of Korea; ^4^Department of Life Sciences, Korea University, Seoul, Republic of Korea

**Keywords:** virtual reality, anxiety, assessment, panic disorder, physiological responses, relaxation, interoceptive exposure, research domain criteria (RDoC)

## Abstract

**Introduction:**

Recurrences and diagnostic instability of panic disorder (PD) are common and have a negative effect on its long-term course. Developing a novel assessment tool for anxiety that can be used in a multimodal approach may improve these problems in panic disorder patients. This study assessed the feasibility of virtual reality-based assessment in panic disorder (VRA-PD).

**Methods:**

Twenty-five patients with PD (ANX group) and 28 healthy adults (CON group) participated in the study. VRA-PD consisted of four modules based on the key components of cognitive behavior therapy for an anxiety disorder: “Baseline evaluation module” (M0), “Daily environment exposure module” (M1), “Relaxation module” (M2), and “Interoceptive exposure module” (M3). Multiple evaluations, including self-rating anxiety scores (AS) and physiological responses [heart rate variability (HRV) index], were performed in three steps at M1, M2, and M3, and once at M0. Comparisons between patients with PD and healthy controls, factor analysis of variables in VRA-PD, changes in responses within modules, and correlation analysis between variables in VRA-PD and anxiety symptoms assessed by psychological scales were performed.

**Results:**

All participants completed the VRA-PD without discontinuation. The ANX group reported significantly higher AS for all steps and a smaller HRV index in M1 (steps 1 and 2) and M2 (step 1). Repeated-measures analysis of covariance (ANCOVA) revealed significant interaction effects for AS in M1 (*F* = 4.09, *p* = 0.02) and M2 (*F* = 4.20, *p* = 0.02), and HRV index in M2 (*F* = 16.22, *p* < 0.001) and M3 (F = 21.22, p = 0.02). The HRV index only indicated a good model fit for the three-factor model, reflecting the construct of the VRA-PD. Both AS and HRV indexes were significantly correlated with anxiety and depression symptoms.

**Discussion:**

The current study provides preliminary evidence that the VRA-PD could be a valid anxiety behavior assessment tool.

## 1. Introduction

Panic disorder (PD) is an anxiety disorder characterized by the occurrence of unexpected panic attacks, in which an overwhelming anxiety accompanied by a sequence of physiological and/or cognitive symptoms emerges suddenly and without an obvious external reason (1). According to epidemiological research, the lifetime prevalence of PD in the general adult population is between 1.4 and 4.1% (2–4). Moreover, recurrences of anxiety disorders are common, with reports of 56 and 58% for PD without agoraphobia and PD with agoraphobia, respectively (5). Recurrences of anxiety disorders have a negative effect on the long-term course of these diseases (6). Therefore, modifying treatment strategies by identifying recurrence-related predictors might contribute to the decrease in recurrence.

Over the last decades, a growing body of research has discovered diagnostic changes within anxiety disorders (2, 6), as well as between depressive and anxiety disorders (7, 8). In addition, it has been reported that such diagnostic instability is related to recurrence (9). These results on diagnostic instability in anxiety disorders are consistent with twin and family research on comorbidity findings, indicating that overlapping genetic etiological components are likely represented as personality characteristic of neuroticism (10). The predictive problem of recurrence and diagnostic instability shows the lack of an evaluation system that relies on expert interviews and self-report in the current anxiety disorder evaluation (11). Moreover, concerning the recent need for precision medicine, categorical diagnostic systems are considered a need for significant improvement in psychiatry related to these issues (12).

In this context, the National Institute of Mental Health has initiated the Research Domain Criteria (RDoC) initiative, which stresses a multimodal approach to identify relationships between neurobiological markers, clinical behaviors, and trait characteristics that span traditional diagnostic categories (13). However, few investigations of anxiety disorders using the RDoC have been described. It may be because behavior-related variables have not yet been well established in the negative valence systems and arousal and regulatory systems; domains associated with anxiety disorder in the RDoC matrix (14). Even in the previous research, although anxiety was not explicitly investigated, the “Emotion” task or “threat conditioning and extinction” task was used as a measure (15, 16). Therefore, developing a tool capable of assessing anxiety-related behavior will play a crucial part in our ability to comprehend and design treatments for those suffering from anxiety.

Virtual reality (VR) may be an advantageous tool for assessing anxiety behaviors because it immerses people in a virtual world that mirrors daily living requirements (17–20). Previous research has shown that VR offers an enormous opportunity to evaluate whether therapies can be applied in real life and can allow scientists to observe an individual’s real-time interactions with virtual entities (21, 22). We also reported that psychological factors, including communication style and life satisfaction, are related to behavior in VR (23, 24). VR can also induce intended anxiety behavior in patients with anxiety disorders, based on the findings that the use of VR is effective for cognitive behavioral therapy for anxiety disorders and the exploring the mechanism of action underlying exposure therapy (25, 26). In addition, providing and observing stimuli in real time is advantageous for applying a multimodal approach, including the simultaneous acquisition of physiological data. Moreover, the use of VR reduces human resources and costs and thus has an advantage in terms of sustainability (27). Therefore, it can be said that VR possesses the necessary qualities for assessing anxiety-related behavior.

In response to this need, we developed virtual reality-based assessments in patient with panic disorder (VRA-PD), an anxiety behavior evaluation system, to evaluate the characteristics of an individual suffering from anxiety. The VRA-PD uses the virtual environment data of the VR-based relaxation self-training program for PD, the feasibility of which has been confirmed in other studies (25). In the VRA-PD, we acquired both subjective anxiety and physiological responses in virtual environments that were developed to induce anxiety-related behaviors. The VRA-PD scenario comprises modules that represent different parts of cognitive behavioral therapy for anxiety disorders. These modules included a claustrophobic and social environment (getting on an elevator), interoceptive sensations (hyperventilation and head down), and relaxation training (28, 29).

The current study aimed to summarize the implementation method of this VRA-PD and evaluate its feasibility in assessing anxiety disorders by applying it to patients with PD and healthy adults without anxiety. By introducing the system configuration of VRA-PD and the content and purpose of its modules, we attempted to demonstrate how virtual reality technology may be used as a tool for assessing mental health. To assess it feasibility, this study explored the discriminant, construct, and convergent validity of VAR-PD in patients with PD. We hypothesized that variables within VRA-PD differ significantly between patients with PD and healthy controls, represent a three-factor model reflecting the construct of VRA-PD, and significantly correlate with anxiety symptoms.

## 2. Materials and methods

### 2.1. Participants

Patients with PD and healthy controls, matched for sex, age, and marital status, were recruited through outpatient clinics and public advertisements. All examinations and procedures were conducted at the psychiatric outpatient clinic of Korea University Guro Hospital. All patients were interviewed by a psychiatrist using the Mini-International Neuropsychiatric Interview (MINI) to screen for histories of psychiatric illness and drug use (30).

The inclusion criteria for patients with PD were as follows: (1) symptoms complied with the diagnostic criteria for PD according to the Structured Clinical Interview of the MINI; (2) age between 19 and 50 years; and (3) voluntary participation with signed informed consent. The exclusion criteria for patients with PD were as follows: (1) nervous system diseases or other mental illnesses, major physical illnesses, or serious infectious diseases; (2) alcohol or substance abuse; and (3) major depressive episodes, bipolar I disorder, or psychotic disorders. The inclusion criteria for healthy controls were as follows: (1) age between 19 and 50 years and (2) voluntary participation with signed informed consent. The exclusion criteria for healthy controls were as follows: (1) suffering from nervous system diseases or other mental illness, major physical illness, or serious infectious diseases; and (2) history of mental illness. The study was approved by the local ethics committee of Korea University Guro Hospitals (2021-GR0057), and each participant signed a written informed consent form after being notified of the objectives, methods, and possible risks of the study. All aspects of the study were conducted in accordance with the 1964 Declaration of Helsinki.

Thirty patients and 30 healthy controls met the eligibility criteria and participated in this study. Seven participants were excluded due to incomplete physiological assessment data. The final sample consisted of 53 adults, representing two groups: 25 adults with PD (ANX group) and 28 healthy adults (CON group). Table 1 shows the demographic characteristics and means assessment scores of the sample.

**TABLE 1:** Demographic and psychological characteristics of each group.

Variable	CON (*n* = 28)		ANX (*n* = 25)		*t/*χ*^2^*	*P*-value
	Mean/n	SD/%	Mean/n	SD/%		
Age (years)	33.11	9.62	34.52	11.33	-0.491	0.626
Sex (female, %)	20	71.4	12	48.0	3.030	0.082
Marital status					3.902	0.272
Never married	20	71.4	14	56.0		
Married	8	28.6	8	32.0		
Separated or divorced or widowed	0	0.0	3	8.0		
Smoking (pack/day)	0.08	0.26	0.30	0.45	-2.176	0.034
Alcohol (glass/week)	6.25	9.77	19.85	28.98	-2.342	0.023
PDSS	-	-	14.08	4.02	-18.555	<0.001
**LSAS-SR**						
Fear	22.25	14.56	28.44	15.74	-1.487	0.143
Avoidance	19.86	12.35	27.20	15.86	-1.891	0.064
GAD-7	3.82	3.49	11.04	5.76	-5.590	<0.001
**HADS**						
Anxiety	4.82	2.76	12.08	5.02	-6.619	<0.001
Depression	6.71	4.04	10.96	3.94	-3.861	<0.001

The statistical value is the *t*-value in an independent *t*-test for continuous data and χ^2^ in the chi-square test for categorical data. SD, standard deviation; PDSS, panic disorder severity scale; LSAS, liebowitz social anxiety scale; GAD-7, generalized anxiety disorder-7; HADS, hospital anxiety depression scale.

### 2.2. System configuration and operation

**FIGURE 1:**
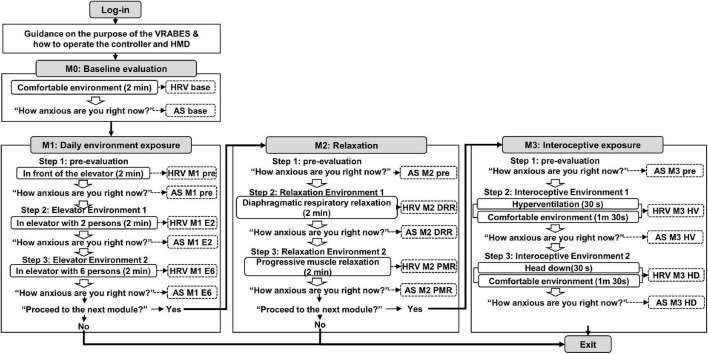
Configuration diagram of the virtual reality-based assessment in panic disorder (VRA-PD). A box with rounded corners inside an angled rectangle representing a module describes the virtual environment. An empty arrow means a process that checks whether to proceed after the virtual environment. Items marked with dotted lines indicate recorded variables. M, module; DRR, diaphragmatic respiratory relaxation; PMR, progressive muscle relaxation; HV, hyperventilation; HD, head down.

This section presents the design of the proposed VRA-PD, as outlined in Figure 1. The VRA-PD scenario was developed by a psychiatrist with experience in developing VR-based programs for psychological interventions and treating patients with anxiety disorders using a VR-based relaxation self-training program. In addition, the scenario and virtual environment were modified through a review by two other psychiatrists who were experts in treating anxiety disorders.

After the login process, voice guidance on the purpose of the VRA-PD and how to operate the controller and head-mounted display (HMD) were provided to the users. Subsequently, users progressed through four consecutive modules; “Baseline evaluation,” “Daily environment exposure,” “Relaxation,” and “Interoceptive exposure.” Except for the baseline evaluation module, the other three modules consisted of three steps as follows: (1) pre-evaluation environment, (2) virtual environment 1, and (3) virtual environment 2. At the end of each module and virtual environment, the decision to proceed to the following module or the following virtual environment was selected.

The virtual environments of the VRA-PD were produced by modifying the virtual environments of the VR-based relaxation self-training program developed in previous research and confirmed to be suitable for treatment (25). Except for the virtual environment of the daily environment exposure module produced with animated graphics, the remaining three virtual environments were created with a 3D video filmed in a real scene using a 360-degree 3D camera (Insta360 Pro, Insta360 Inc., Irvine, CA, USA).

The virtual environments were exhibited using an HMD comprising an Oculus Quest 2 and two Oculus Touch Controllers to provide a 360-degree view with an 89-degree field of vision. Users may independently execute VRA-PD based on the built-in instructions provided as text on the screen or audibly through the audio system. Furthermore, Quest 2 and the physiological data acquisition system were connected to the same laptop to interlock the physiological data and VRA-PD. The researcher checked the progress through a laptop screen to monitor the appropriate process. Users can proceed with all VRA-PD processes by clicking icons on the screen with a spear-shaped pointer using the controller. When it was no longer feasible to continue experiencing the virtual environment due to cybersickness or rising anxiety, the user was notified that the evaluation could be terminated by removing the HMD.

### 2.3. In-app measurements

**FIGURE 2:**
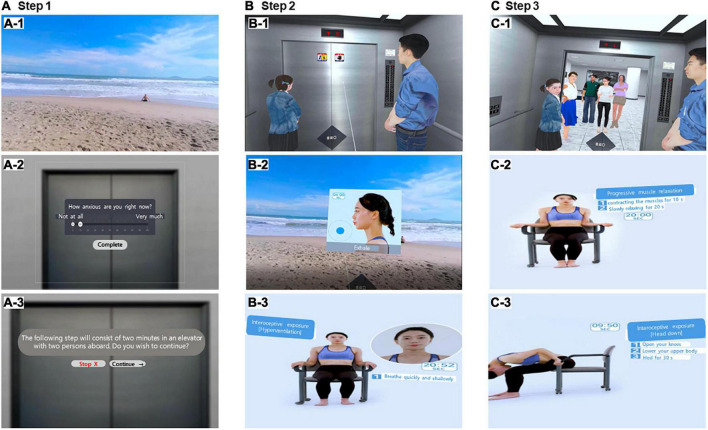
Screenshots of the virtual reality-based assessment in panic disorder (VRA-PD). In the program, Modules 1, 2, and 3 consisted of three steps (pre-evaluation, step 1, and step 2). Module 0 (baseline evaluation module) with a comfortable virtual environment composed of beach scenes **(A-1)**. A comfortable virtual environment **(A-1)** was employed in step 1 of Module 1. In addition, it was also used in Module 3 at 1 min and 30 s in the second half of the interoceptive exposure of Step 2 and Step 3. Users rate their self-rating anxiety scores on the visual analog scale constructed in the virtual environment **(A-2)**. Users selected whether to proceed at each step and each module **(A-3)**. In Module 1, Steps 2 **(B-1)** and 3 **(C-1)** each consist of 2 min process in an elevator with two and six passengers, respectively. Users performed diaphragmatic respiratory relaxation (DRR) in step 2 **(B-2)** of Module 2 and progressive muscle relaxation (PMR) in step 3 **(C-2)** for 2 min each according to human motion, guidance, and time displayed in the virtual environment. Panel **(B-3)** shows the hyperventilation virtual environment of module 3, and panel **(C-3)** shows the head down virtual environment. In Module 3, the guidance in the virtual environment was used in the same manner as in Module 2 for 30 s to provide stimulation. Panel **(A-1)** shows the comfortable scene for the remaining 1 min and 30 s. The program was conducted in Korean; however, the examples of the help balloon in this figure are presented in English to aid the understanding of readers.

Subjective VRA-PD anxiety experiences and physiological data were collected in the VRA-PD. Evaluation of subjective anxiety experience (anxiety scores, AS) was based on the question, “How anxious are you right now?” The participants’ responses were rated on a visual analog scale (VAS), which presented “not at all” (0 points) at the left end of a horizontal line and “very much” (100 points) at the right end (Figure 2A-2). Subjective anxiety experience was recorded as AS, and the time at which ASs were received for each module and the variable names are shown in Figure 1.

Physiological data, composed of photoplethysmogram (PPG) data, were measured for 2 min in each virtual environment. As shown in the overall system configuration (Figure 1), data were collected once in the baseline evaluation module, thrice in the daily environment exposure module, and twice in the other two modules. PPG data were measured using a PPG sensor [Model: ubpulse T1 (Pulse Analyzer, MFDS, Certification No. 11-1296), LAXTHA Inc., Daejeon, South Korea], approved as a medical device by the Ministry of Food and Drug Safety of South Korea. Ubpulse T1 has a sampling rate of 250 Hz and a bandpass frequency of 0.3–10.6 Hz.

A PPG sensor was placed on the index finger of the left hand, and the hands connected to the sensors were placed on the armrest of a chair and relaxed. Manicured fingernails or foreign substances were removed from the fingernails attached to the pulsing electrode. In addition, tight-fitting sleeves, disposable bands, and rubber bands that exerted pressure on the arms or fingers were removed.

The 2-min PPG measurement period may not be adequate to ensure repeatability (31). In addition, artifacts induced by user movement due to the nature of the VR environment may impact data quality. The PPG parameter employed in this research was the heart rate variability (HRV) triangular index (HRV index). The HRV index is based on geometric approaches insensitive to data quality and demonstrates robustness to outliers and artifacts (32, 33).

Therefore, the heart rate variability triangular index (HRV index) was employed among the parameters obtained by analyzing PPG because, the HRV index has been examined across anxiety disorder patient groups, including those with PD (34–36), and its predictive value in heart-related disorders is also established (37). The analysis for the HRV index was conducted based on standard methods (38).

### 2.4. Contents of each module

#### 2.4.1. Baseline evaluation module (module 0)

The baseline evaluation module was designed to assess the baseline state before exposure to the virtual environment of the module for participant evaluation and to introduce the evaluation method repeatedly used in each module. Users were instructed to rest for 2 min in a comfortable virtual environment composed of a beach scene with a clear sky (Figure 2A-1). In this module, physiological data were measured while experiencing a comfortable virtual environment, and the participants’ subjective anxiety experience was evaluated afterward.

#### 2.4.2. Daily environment exposure module (module 1)

**FIGURE 3:**
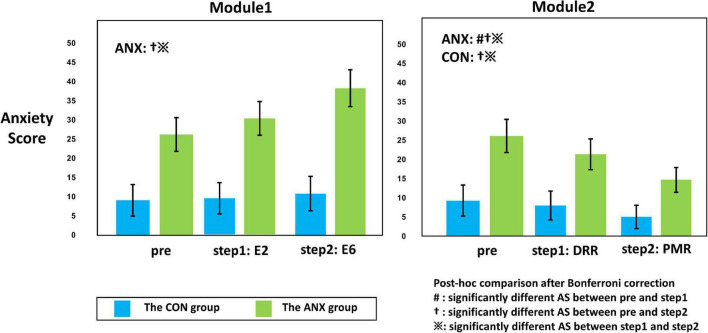
Illustration of the interaction effect of group (ANX and CON) x time (pre, step 1, and step 2) obtained from repeated-measured analysis of covariance (ANCOVA). The adjusted value for sex, age, smoking usage, alcohol usage, and HADS-D are presented for each module. Error bars mean standard errors. The *post hoc* comparison of the significant ANCOVA results is also described within the plot. The symbols in the graph mean significantly different self-rating anxiety scores between pre and during (#), between pre and post (y), and between during and post (※) from the *post hoc* comparisons with Bonferroni-corrected *p* < 0.05. AS, anxiety scores; DRR, diaphragmatic respiratory relaxation; PMR, progressive muscle relaxation.

The daily environment exposure module was designed to assess anticipatory anxiety in daily environments and changes in anxiety according to changes in stress intensity. This virtual environment was designed with an elevator boarding environment, one of the ordinary daily life situations precipitating anxiety reported in a previous study using VR in patients with PD (39). The daily environment exposure module consisted of three virtual environments: (1) evaluation of anticipatory anxiety standing in front of the elevator; (2) elevators in which two passengers rode together; (3) elevators in which six passengers rode together. When the users selected the Module “Daily environment exposure” among the four icons shown after Module 0, the screen was converted into a virtual environment standing in front of the elevator entrance in the building (Figure 2A-2). In the evaluation of anticipatory anxiety, users were instructed to imagine the scene of the boarding elevator with two and six passengers for 2 min. Subsequently, two-stage virtual environments were conducted in which two and six passengers boarded the elevator together in order for 2 min each (Figures 2B-1, 2C-1). Physiological data acquisition during each virtual environment for 2 min and subjective AS ratings after each environment were performed three times for each virtual environment (Figures 2A-3).

#### 2.4.3. Relaxation module (module 2)

The relaxation module was designed to evaluate the relaxation ability of patients with anxiety disorders. This module was designed to sequentially experience two virtual environments associated with diaphragmatic breathing relaxation and progressive muscle relaxation, which are essential elements of cognitive behavioral therapy (40). When the user finished the daily environment exposure module and pressed the “Next” icon, the screen was converted into the beach virtual environment screen used in the baseline evaluation module. They listened to the voice guidance about the module’s objectives and content, and the subjective AS was rated. Notably, physiological data were not acquired in this scene. In a virtual environment related to diaphragmatic breathing relaxation and progressive muscle relaxation, users were instructed to relieve tension by diaphragmatic breathing or forcefully contracting the muscles for 10 s and then slowly relaxing them for 20 s. Users underwent relaxation according to the help video of the assistant’s motion played in the center of the screen for 2 min (Figures 2B-2, 2C-2). Physiological data acquisition and subjective AS ratings were performed twice for each virtual environment.

#### 2.4.4. Interoceptive exposure module (module 3)

The interoceptive exposure module was designed to evaluate the sensitivity to interoceptive stimuli related to anxiety disorders. Users who chose to proceed from the relaxation module to the interoceptive exposure module were instructed by the voice guidance for the interoceptive exposure module. They proceeded with the pre-state self-rating anxiety evaluation in the same way as the relaxation module. Afterward, users experienced two virtual environments sequentially as the video of the assistant’s motion played in the center of the screen for hyperventilation (HV) and head down (HD), respectively (Figures 2B-3, 2C-3). Because of the difficulty of performing interoceptive stimuli and the risk of inducing panic attacks, both virtual environments performed HV and head down for the first 30 s out of 2 min in the production process. The remaining 1 min and 30 s were designed to look at the comfortable environment of the beach. Physiological data acquisition and subjective AS ratings were performed twice for each virtual environment.

### 2.5. Psychological assessments

#### 2.5.1. Interview measures

The panic disorder severity scale (PDSS) is a seven-item, clinician-administered standard assessment of PD and agoraphobia symptom severity (41). This study used the Korean version of the PDSS, translated and standardized in 2001 (42). Five PDSS questions were used to evaluate the basic DSM-IV symptoms of PD, while two additional measures were for assessing impairment in social and occupational functioning. Each item was graded on a scale from 0 to 4 for a total of 28 points. The PDSS has demonstrated sound psychometric properties with good internal consistency (Cronbach’s α = 0.88) and test-retest reliability (*r* = 0.71) (43). The Cronbach’s α for the ANX group in this study was 0.86.

#### 2.5.2. Liebowitz social anxiety scale: Self-report version

The Liebowitz social anxiety scale: self-report version (LSAS-SR) is a self-reported version of the LSAS (44), a 24-item semi-structured interview assessment of fear and avoidance in various social and performance circumstances. Participants were given a copy of the questionnaire and were asked to score each item twice, based on how much they dreaded the circumstance and then on how often they avoided it. The psychometric qualities of the LSAS-SR were presented with good internal consistency (Cronbach’s α = 0.95) and test-retest reliability (*r* = 0.70) (45). Each of the 24 events was assessed on a 4-point scale (0–3), first for fear and then for avoidance. Zero represents no fear, one represents mild fear, two represents considerable fear, and three represents extreme terror. In addition, zero indicates no avoidance, one indicates occasional avoidance (1–33 percent of the time), two indicates often avoiding the circumstance (34–67 percent of the time), and three indicates always avoiding the situation (68–100 percent of the time). The Korean version of the LSAS-SR, adapted and validated by Kang et al. (46) in 2013, was used. In the current study, Cronbach’s alpha of the LSAS-SR-fear and LSAS-SR-avoidance was 0.94 and 0.95, respectively, indicating an acceptable level of internal consistency.

#### 2.5.3. Generalized anxiety disorder scale

The generalized anxiety disorder scale (GAD-7) is a 7-item self-reported instrument designed to screen for GAD symptoms (47). Participants were asked how often they had been disturbed by anxiety symptoms (such as difficulty relaxing or excessive concern) over the previous 2 weeks. On a 4-point Likert-type scale, items are ranked from 0 (not at all) to 3 (almost every day). Higher scores indicate more severe symptoms of GAD. In primary care settings and with the general population, the GAD-7 has been proven to have sufficient psychometric qualities, including strong internal consistency (Cronbach’s α = 0.92), clinical value, and construct validity (48, 49). This study used the Korean version of the GAD-7 with confirmed psychometric qualities (50). The GAD-7 also showed good reliability in the current study (Cronbach’s α = 0.93).

#### 2.5.4. Hospital anxiety and depression scale

The hospital anxiety and depression scale (HADS) consists of two subscales: the HADS-A, designed to detect anxious states, and the HADS-D, designed to detect depressive states (51). Each subscale consisted of seven items with a 4-point ordinal response format. Scores ranged from 0 to 21 for each subscale, with higher scores indicating higher levels of anxiety or depression. Participants answered each item by thinking about how they felt and/or behaved during the past week. In this study, the Korean versions of the HADS with good internal consistency [Cronbach’s α = 0.89 (HADS-A) and 0.86 (HADS-D)] were used (52). Cronbach’s α of the HADS-A and HADS-D was 0.94 and 0.84, respectively, indicating high levels of internal consistency in the current study.

#### 2.5.5. Simulator sickness questionnaire

The simulator sickness questionnaire (SSQ), a 16-item questionnaire, was used to examine the occurrence and severity of cybersickness symptoms when immersed in virtual environments (53). The SSQ has been extensively used to assess and minimize simulator sickness and to investigate any significant impacts (e.g., age, gender, and equipment characteristics) on simulator sickness (54). Cronbach’s α for the Korean version of the SSQ was 0.89, indicating good internal consistency (55). The SSQ also showed good reliability in the current study (Cronbach’s α = 0.93).

### 2.6. Statistical analyses

All statistical analyses were performed using a commercial software package (IBM SPSS Statistics v28.0, AMOS v.28.0, for Windows, IBM Corporation, Armonk, NY, USA). Demographic variables, and psychological assessments were compared between the ANX and CON groups using an independent *t*-test for continuous data and the chi-square test for categorical data. Distributions of the subjective AS and physiological measures of the VRA-PD were presented with corresponding item skewness and kurtosis values and then compared between the two groups using the Mann–Whitney test. A repeated-measures analysis of covariance (ANCOVA) was performed to evaluate content validity of the VRA-PD, controlling for age, sex, smoking usage, alcohol usage, and HADS-D scores. Since the difference in the subjective AS between the two groups was significant, both the group factor and the repetition effects were included. For physiological data, repeated-measures ANCOVA for only the repetition effect was executed only in the ANX group, a target group. Next, the VRA-PD construct validity for each acquired variable was examined to show whether the variables in the VRA-PD fit well by module. To validate the factor structure of the VRA-PD, we employed confirmatory factor analyses with maximum likelihood implemented in AMOS v.28.0 concerning each variable in VRA-PD to account for probable non-normality in the data distribution. We tried a three-factor model in which the variables from each module were added to the module factor because each module provides a different stimulus. Model fit was assessed using the chi-squared statistic, comparative fit index (CFI), Tucker–Lewis index (TLI), root mean square error of approximation (RMSEA), and standardized root mean squared residual (SRMR). Although a consensus on acceptable levels of fit indices is still missing (56), a value of 0.95 or more for CFI and TLI, 0.08 or less for RMSEA, and 0.08 or less for the SRMR is considered an acceptable fit (57). Concerning convergent validity, we analyzed correlations among psychophysiological measures in the VRA-PD with psychological assessments concerning anxiety PDSS, LSAS-SR, GAD-7, HADS-D, and SSQ using Spearman’s rank correlation analysis. Statistical significance was set at *p* < 0.05.

## 3. Results

### 3.1. Demographic and self-assessment scales

Statistical tests did not show significant differences in age, years of education, sex, and marital status distribution between the two groups. The ANX group presented more nicotine and alcohol use than the CON group (Table 1). A significant difference was found in the psychological characteristics associated with anxiety, except for the LSAS-SR-fear and LSAS-SR-avoidance subscale scores.

### 3.2. Acceptability of VRA-PD

Participants in the ANX and CON groups did not discontinue the VRA-PD because of anxiety symptoms, including omitted cases due to inadequate data quality. Cybersickness measured by the SSQ after VRA-PD use was more significant in the ANX group (mean = 34.68, SD = 12.46) than in the CON group (mean = 21.93, SD = 8.21; *t*_51_ = −4.44, *p* < 0.001).

### 3.3. Distribution of variables in VRA-PD and discriminant validity

Table 2 presents the distribution and descriptive statistics of AS and HRV index of each module with means, standard deviations, median, skewness, and kurtosis concerning ANX and CON groups. The median of all ASs in the CON group was 0, and most of the scores had skewness of more than two, indicating that it was not a normal distribution (58). The skewness and kurtosis values of ASs from the ANX group were within the acceptable range, considering they are a normal distribution. The ANX group showed greater self-rating AS than the CON group for all ratings concerning each module except for baseline AS.

**TABLE 2:** Distribution and descriptive statistics of the psychophysiological data in each module.

Variables	CON (*n* = 28)					ANX (*n* = 25)					Mann–Whitney *z*	*P*-value
	Mean	SD	Median (IQR)	sk	ku	Mean	SD	Median (IQR)	sk	ku		
**Self-rated anxiety**												
Baseline	13.21	23.89	0 (17.5)	2.19	3.85	26.8	32.11	10 (55.0)	1.16	0.2	1.900	0.057
M1 pre	5.36	12.01	0 (10.0)	3.78	16.59	30.4	27.31	30 (35.0)	1.1	0.82	4.391	<0.001
M1 E2	6.07	12.57	0 (10.0)	3.35	12.95	34	27.08	30 (40.0)	0.69	-0.04	4.408	<0.001
M1 E6	6.79	12.78	0 (10.0)	2.95	10.82	43.2	30.51	30 (50.0)	0.43	-0.9	4.991	<0.001
M2 pre	5.36	12.32	0 (10.0)	3.56	14.78	33.6	28.71	30 (40.0)	0.72	-0.52	4.497	<0.001
M2 DRR	4.29	10.34	0 (7.5)	3.56	14.62	25.6	26.47	20 (40.0)	1.19	1.06	4.010	<0.001
M2 PMR	2.5	7.99	0 (0)	4.19	19.17	17.6	21.66	10 (25.0)	1.51	1.82	3.797	<0.001
M3 pre	4.64	9.62	0 (7.5)	2.4	6.06	25.6	28.3	20 (35.0)	1.31	0.83	3.795	<0.001
M3 HV	6.43	11.62	0 (10.0)	1.69	1.78	25.6	24.51	20 (40.0)	0.93	0.3	3.520	<0.001
M3 HD	3.93	10.31	0 (0)	3.7	15.43	22.4	27.58	10 (30.0)	1.34	0.59	3.592	<0.001
**HRV index**												
Baseline	8.20	1.82	8.09 (3.25)	0.33	-0.31	7.60	2.49	7.35 (3.45)	0.38	-0.41	-1.025	0.306
M1 pre	8.59	2.54	8.27 (3.47)	0.74	0.78	7.43	2.12	6.95 (3.81)	0.57	-0.25	-1.693	0.091
M1 E2	8.93	1.66	8.92 (3.26)	0.15	-1.41	7.06	2.22	6.68 (2.47)	2.37	8.23	-3.858	<0.001
M1 E6	8.49	1.85	8.30 (2.84)	-0.30	-0.12	7.33	2.72	7.00 (3.55)	1.49	3.91	-2.432	0.015
M2 DRR	13.05	2.69	12.57 (3.46)	1.21	1.42	10.78	3.11	10.11 (4.24)	0.31	-0.17	-2.824	0.005
M2 PMR	10.14	3.32	8.88 (5.32)	0.90	-0.06	9.50	3.45	9.05 (4.94)	0.65	-0.03	-0.748	0.454
M3 HV	7.33	2.94	6.28 (3.36)	1.69	3.19	5.88	1.62	5.83 (1.29)	0.81	1.16	-1.667	0.096
M3 HD	7.33	2.87	6.57 (4.15)	1.41	2.73	6.31	1.85	6.60 (2.72)	0.52	0.75	-1.114	0.265

Values are displayed as mean, standard deviation, and median (interquartile range). CON and ANX were compared using the Mann–Whitney test. IQR, interquartile range; sk, skewness; ku, kurtosis.

The HRV index was significantly lower in the ANX group elevators 2 and 6 of module 1 (M1) and in the diaphragmatic respiratory relaxation (DRR) environment of module 2 (M2) than in the CON group (M1 elevator 2: Mann–Whitney *U* = −3.86, *p* < 0.001; M1 elevator 6: Mann–Whitney *U* = −2.43, *p* = 0.015; M2 DRR: Mann–Whitney *U* = −2.82, *p* = 0.005).

### 3.4. Content validity

#### 3.4.1. Self-rating anxiety scores

For the repeated measured ANCOVA results of AS for each module (Table 3), a significant interaction effect between group (ANX vs. CON) and time (pre vs. during vs. post) was found for M1 and M2, rather than for the AS of Module 3 (M3). For all modules, a significant main effect of the group was shown, and the *post hoc* test showed higher AS in the ANX group than in the CON group. A significant main effect of time was revealed for MI.

**TABLE 3:** Results of repeated-measured ANOVA for self-rating anxiety scores in the two groups (ANX and CON) and different time concerning each module.

	Main effect-group		*Post hoc*	Main effect-time		*Post hoc*	Interaction effect		*Post hoc*
	*F*	*P*-value		*F*	*P*-value		*F*	*P*-value	
Module 1	11.373	0.002	CON < ANX	4.239	0.017	pre < E6 E2 < E6	4.085	0.02	See Figure 3
Module 2	6.736	0.013	CON < ANX	0.474	0.624		4.198	0.018	See Figure 3
Module 3	5.24	0.027	CON < ANX	0.225	0.799		0.061	0.941	

Figure 3 shows the significant results among the paired *t*-tests for each group, conducted as a *post hoc* test for the interaction effect shown in M1 and M2. In the CON group, there was no significant change in AS at each measurement time point (pre vs. E6: *t_27_* = 1.28, Bonferroni-corrected *p* = 0.424; pre vs. E2: *t*_27_ = 1.00, Bonferroni-corrected *p* = 0.652; E2 vs. E6: *t_27_* = 0.70, Bonferroni-corrected *p* = 0.980) in M1, and AS M2 progressive muscle relaxation (PMR) was significantly decreased compared to AS M2 pre- and AS M2 DRR (pre vs. PMR: *t_27_* = −2.83, Bonferroni-corrected *p* = 0.018; DRR vs. PMR: *t_27_* = −2.42, Bonferroni-corrected *p* = 0.044) in M2.

In the ANX group, AS M1 E6 was significantly increased compared to AS M1 pre and AS M1 E2 (pre vs. E6: *t_24_* = 3.251, Bonferroni-corrected *p* = 0.006; E2 vs. E6: *t_24_* = 3.258, Bonferroni-corrected *p* = 0.006). In M2, AS decreased significantly from pre-to DRR and PMR (pre vs. DRR: *t_24_* = −2.954, Bonferroni-corrected *p* = 0.014; pre vs. PMR: *t_24_* = −4.899, Bonferroni-corrected *p* < 0.001; DRR vs. PMR: *t_24_* = −3.024, Bonferroni-corrected *p* = 0.012).

#### 3.4.2. Physiological data

Regarding the HRV index, a significant repetition effect was found for M2 (*F*_2_,_22_ = 16.21, *p* < 0.001) and M3 (*F*_2_,_22_ = 21.22, *p* < 0.001), rather than for M1 (*F*_2_,_22_ = 1.15, *p* = 0.34). The HRV-index significantly increased compared to E6 in both DRR and PMR in M2 (E6 vs. DRR: *t_24_* = 6.07, Bonferroni-corrected *p* < 0.001; E6 vs. PMR: *t_24_* = 3.39, Bonferroni-corrected *p* = 0.001). In M3, both HV and head down (HD) decreased significantly compared with PMR (PMR vs. HV: *t_24_* = −5.35, Bonferroni-corrected *p* < 0.006; PMR vs. HD: *t_24_* = −4.42, Bonferroni-corrected *p* < 0.006).

### 3.5. Factor structure, confirmatory factor analysis

**FIGURE 4:**
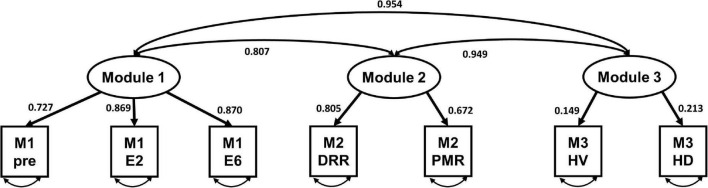
The standardized model of the three-factor heart rate variability (HRV) index of virtual reality-based assessment in panic disorder (VRA-PD). M, module; DRR, diaphragmatic respiratory relaxation; PMR, progressive muscle relaxation; HV, hyperventilation; HD, head down.

Table 4 presents the CFA results without correlating the error terms for each variable in the total sample. Regarding the fit statistics, the results for the HRV index only indicated a good model fit, with chi-square statistics (χ2 = 391.92, χ2/df = 48.99), RMSEA = 0.026, CFI = 0.949, TLI = 0.903, and SRMR = 0.063. Figure 4 shows the standardized estimates of the confirmatory model and the correlations among the modules.

**TABLE 4:** Confirmatory factory analysis of self-rating anxiety score and heart rate variability (HRV) index in virtual reality-based assessment in panic disorder (VRA-PD).

Variables	χ^2^	df	χ^2^/df	RMSEA (90% CI)	CFI	TLI	SRMR
AS	85.503	24	3.479	0.218 (0.168–0.270)	0.917	0.875	0.040
HRV index	17.162	11	1.560	0.026 (0.000–0.184)	0.949	0.903	0.063
Acceptable cut-off value			<3	<0.06	>0.90	>0.90	<0.08

CFI, comparative fit index; TLI, Tucker–Lewis index; RMSEA, root mean square error of approximation; SRMR, standardized root mean squared residual.

### 3.6. Convergent validity

Table 5 presents the results of the Spearman correlation analysis. Several HRV indexes exhibited substantial relationships with anxiety-related PDSS, LSAS-SR, and GAD-7. The HRV index was also significantly correlated with depression-related HADS-D rather than with the SSQ. AS exhibited a strong association with anxiety-related measures. There was also a strong association between AS, HADS-D, and SSQ.

**TABLE 5:** Correlations among variables in virtual reality-based assessment in panic disorder (VRA-PD), psychological characteristics and simulator sickness questionnaire (SSQ) in the ANX group.

	PDSS	LSAS-SR -fear	LSAS-SR -avoidant	GAD-7	HADS-D	SSQ
**HRV index**						
Base	0.198	0.459*	0.556**	0.507**	0.387	0.211
M1 pre	0.127	0.319	0.363	0.525**	0.574**	0.322
M1 E2	0.194	0.272	0.350	0.456*	0.452*	0.055
M1 E6	0.235	0.382	0.428*	0.405*	0.338	-0.005
M2 DRR	-0.009	-0.182	-0.012	0.216	0.174	-0.238
M2 PMR	-0.005	0.291	0.333	0.483*	0.441*	0.162
M3 HV	-0.433*	-0.228	-0.094	-0.122	0.063	-0.069
M3 HD	0.313	0.086	0.168	0.080	0.018	0.310
**Self-rating anxiety scores**						
Base	0.332	0.351	0.354	0.408*	0.376	0.203
M1 pre	0.056	0.367	0.406*	0.702**	0.509**	0.387
M1 E2	0.266	0.490*	0.496*	0.695**	0.460*	0.545**
M1 E6	0.396	0.604**	0.553**	0.656**	0.476*	0.561**
M2 pre	0.353	0.519**	0.480*	0.760**	0.612**	0.490*
M2 DRR	0.240	0.519**	0.490*	0.728**	0.639**	0.494*
M2 PMR	0.415*	0.664**	0.671**	0.676**	0.652**	0.614**
M3 pre	0.351	0.589**	0.556**	0.662**	0.495*	0.530**
M3 HV	0.229	0.634**	0.683**	0.568**	0.556**	0.416*
M3 HD	0.254	0.499*	0.506**	0.461*	0.474*	0.379

The correlation coefficient (*r_s_*) and *p*-values were calculated using Spearman’s rank correlation analysis. The statistically significant results are indicated in bold. *p < 0.05; **p < 0.001. PDSS, panic disorder severity scale; LSAS, liebowitz social anxiety scale; GAD-7, generalized anxiety disorder-7; HADS, hospital anxiety depression scale; SSQ, simulator sickness questionnaire.

## 4. Discussion

The current study explored the feasibility of a VR program for evaluating anxiety behavior in patients with PD. The VRA-PD execution was conducted with patients with PD and healthy adults to identify the reliability and validity of the VRA-PD on the anxiety-related behaviors of PD. The results suggested that the VRA-PD can potentially be a reliable and valid research tool.

Participants were recruited from among volunteers with PD and those without any psychiatric, nervous, or medical illnesses to clarify the differences in anxiety symptoms. There was no significant difference in the demographic characteristics between the two groups; however, there was a significant difference in the usage of alcohol and smoking. Alcohol consumption and smoking have been linked to increased anxiety disorders, including PD (59, 60). As previous studies have shown that the use of substances can affect physiological data (61), these differences support the inclusion of smoking and alcohol use as covariates in the analysis.

Concerning the psychological characteristics of the recruited subjects, in the CON group, the mean PDSS scores were 0 because they had not experienced any panic attacks; however, the mean PDSS scores of the ANX group were 14.08, which is interpreted as markedly ill (62). In the case of LSAS, there was no difference between the two groups in both subscales of fear and avoidance, whereas, in the GAD-7, HADS-A, and HADS-D, it was confirmed that the ANX group was significantly higher than the CON group. Unlike PDSS and LSAS, which are disease-specific, GAD-7 and HADS-A are mainly used as screening tools. This difference seems to have been confirmed, as it is known that specificity is poor for a detailed diagnosis of anxiety disorders (48, 63). People with PD are known to have a relatively high level of depression (64); therefore, it is reasonable to include the HADS-D as a covariate when looking at how depression affects anxiety and other physiological data.

Regarding acceptability, our sample of 53 participants, including 25 patients with PD and 28 healthy adults, completed all the modules in the VRA-PD without discontinuing. Given that the weighted mean SSQ score of the projection type of the head-mounted display used in the previous study was 29.9 (65), the mean SSQ score of 21.93 of the CON group suggests that VRA-PD causes an acceptable level of adverse effects of cybersickness. Previous studies have suggested that anxiety symptoms can mediate cybersickness in a VR environment (66), and the head down also increases cybersickness in highly stressed people (67). Therefore, the significantly high mean SSQ scores of the ANX group could be caused by interoceptive stimuli in the interoceptive exposure module and anxiety symptoms of PD. Additionally, compared to the 36.27 SSQ mean scores reported in a previous VR exposure study in patients with phobia (68), the 34.68 SSQ mean score in the ANX group was considered acceptable.

The median self-rating AS obtained from the VRA-PD was 0 for most items in the CON group and was significantly higher in the ANX group than in the CON group. Inducing subjective anxiety was significantly different in patients with the PD compared with the CON group while causing an acceptable level of anxiety in the ANX group. This result shows that using VRA-PD in patients experiencing anxiety could differentiate the two groups. These results support both its validity and acceptability. In addition, the distribution, skewness, and kurtosis values in the ANX group, unlike the CON group, were within the range of the standard for the normal distribution (58), which also supports the feasibility of applying VRA-PD to patients with PD. Moreover, our results support the use of subjective discomfort in the virtual environment utilization evaluation of PD patients. The subjective unit of discomfort score is included in the self-report unit of analysis of the “acute threat” sub-construct of the “Negative valence system” domain in the RDoC framework. Therefore, it will be possible to use the subjective anxiety scores in the virtual environment we used in future RDoC framework studies.

Regarding physiological data, the HRV index in the ANX group was lower in the two elevator virtual environments of M1 than in the CON group. Even in measures with no significant difference, the HRV index generally tended to be low in the ANX group. Even though there has not been a study of physiological data analysis using a virtual environment in PD, the results of this study are consistent with the results of previous studies— the reduced HRV index in patients with anxiety disorders (69).

Regarding the factorial construct evidence of validity, our data, obtained *via* confirmatory factor analyses, showed that the hypothesized model was only suitable for the HRV index. This result seems to support the idea that the modules of VRA-PD induce different physiological responses, as intended for the HRV index. It seems that the self-rating AS did not show validity because the current VRA-PD measured self-rating anxiety as a VAS score for overall anxiety, which is insufficient to show the differences between the modules. Previous research on dysfunctional communication using VR has shown that the differences between modules are well reflected when specific subjective responses according to the contents of the virtual environment are acquired (23). Therefore, it would be necessary to prepare questionnaires and ratings that can reflect the characteristics of the module to increase the feasibility of the self-rating behavior parameter.

In the repeated-measures ANCOVA, the self-rating AS results were intended to evaluate the content validity of each VRA-PD module. The interaction effect of group and time was confirmed in M1 and M2. In the *post hoc* test, subjective anxiety in the ANX group increased with time in M1 and decreased in M2. Because M1 was designed to make the user feel anxiety gradually and M2 was meant to relax the user repeatedly, the change in the subjective AS shows that each part of the VRA-PD worked as intended. The insignificant result of this change in M3 indicated that the difference between the two types of interoceptive exposure might not be significant. Moreover, a short relaxation period can affect anxiety (70), and there is also the possibility that the comfortable environment shown in each of the two interoceptive exposure virtual environments affects their response.

Among the physiological responses of the VRA-PD modules, significant changes were observed in M2 and M3 concerning the HRV index. In detail, the HRV index increased compared to pre in the relaxed environment of M2 and decreased compared to pre in the exposure environment of M3. Previous research has revealed that anxious individuals have a lower HRV index than healthy individuals (69); hence, this finding can be interpreted as indicating that the VRA-PD relaxation environment induces a relaxed state in the user, while M3 induces a tense state. Therefore, it can be considered that VRA-PD also induces some valid changes in the physiological domain.

The physiological data showed significant changes in the HRV index in M3, unlike in the self-rating AS. These results suggest that the virtual environment of M3 is insufficient to affect subjective anxiety but is sufficient to induce physical changes. This interpretation is consistent with the results of the current study that no change was observed in the physiological data in M1, even though the self-rating AS changed significantly. In anxiety disorders, both the cognitive domain and physiological responses are important (28). When developing an RDoC study, assessing and integrating a variety of both biological and behavioral measures simultaneously are recommended (14). Therefore, our findings are interesting because they show the possibility that behavior and biological signals are acquired simultaneously through a virtual environment and classify users’ cognitive and physical thresholds concerning anxiety. Follow-up studies introducing the RDoC framework are required to establish this possibility, including developing virtual environments related to the paradigms included in RDoC.

Significant correlations were confirmed between VRA-PD variables and anxiety-related measures in the self-rating ASs and physiological variables. This result suggests that the users’ subjective and physical responses in the VRA-PD environment reflect the psychological aspects of their anxiety and show convergent validity related to anxiety. The HADS-D and SSQ also demonstrated a significant correlation between the HRV index measured in VRA-PD and a part of AS, which shows that the measurement in VRA-PD is not specific to anxiety, contrary to expectations. However, this result may also reflect that depression is a significant factor affecting anxiety, and cybersickness is a significant element in the stress experienced in a VR environment (7, 66). Therefore, to obtain a more precise conclusion on convergence validity, it will be necessary to analyze the correlation of factors unrelated to anxiety and cybersickness in future studies. Moreover, for VRA-PD to be clinically valid, a study that directly compares different types of anxiety disorder diagnoses is required.

This study has some limitations. First, our study evaluated behavioral parameters, physiological data, and anxiety-related data in patients with PD and in healthy controls. Results based solely on panic disorder patients may not generalize to a different diagnosis in anxiety disorders. The second limitation of this research is the extent of the validation evidence (i.e., concept, content, and concurrent validity) and the absence of reliability estimations. Using such restricted metrics does not provide a comprehensive scope of evidence for the VRA-PD. The third limitation in our study was the relatively small sample size for each group. Therefore, we need to carefully interpret the results and secure a larger sample size for future studies. A fourth limitation is that we did not include psychoactive drugs usage in the analysis. Because psychoactive drug use can affect the patient’s physiological response, the patient’s response to anxiety disorders may be masked. Our fifth limitation was the lack of assessments concerning participants’ movement and measurement time while acquiring physiological data. Even a tiny movement can change the quality of the physiological data (71), so it is essential to reduce measurement errors by controlling motion-related parameters. Finally, the 2 min measurement time of this study can have affected the quality of physiological data. Previous studies have shown that a 5-min measurement time is needed to enhance the repeatability and reduce uncertainty of measurements (31). However, previous studies also used 2 min for physiological data acquisition (72), and the need to secure acceptability for actual clinical application of VRA-PD supports a measurement time of 2 min. Therefore, additional considerations regarding optimal measurement time and motion parameter correction are required in future studies.

This study provides preliminary evidence and considerations for the future use of VR in anxiety behavior assessments. It also showed that VRA-PD could induce intentional subjective anxiety and physiological responses in patients with PDs. In addition, the variables obtained from the VRA-PD could reflect the differences in both the cognitive and physiological domains of anxiety behavior between PD and healthy controls. Additionally, our results showed the validity of the modular construction of the VRA-PD and its concurrent validity with other anxiety assessment tools. For VRA-PD to be used as an evaluation tool for anxiety behavior, a future study using a multimodal approach, including other biosignals and brain images, is required for patients with more diverse anxiety disorders. However, given the potential for practical advantages of VR formats and the importance of a dimensional approach, we believe that this study should excite scholars and practitioners.

## Data availability statement

The raw data supporting the conclusions of this article will be made available by the authors, without undue reservation.

## Ethics statement

The studies involving human participants were reviewed and approved by the Ethics Committee of Korea University Guro Hospitals. The patients/participants provided their written informed consent to participate in this study.

## Author contributions

B-HK, J-JK, and JK: conceptualization. S-HK, CH, H-GJ, and M-SL: participant evaluation and data acquisition. B-HK and JK: formal analysis and writing—original draft preparation. JK: writing—review, editing, and supervision. All authors contributed to the manuscript and approved the submitted version.
